# Mitochondrial DNA sequence of *Conus textile* (Neogastropoda: Conidae)

**DOI:** 10.1080/23802359.2016.1192513

**Published:** 2016-07-12

**Authors:** Po-Wei Chen, Sheng-Tai Hsiao, Kao-Sung Chen, Chen-Te Tseng, Wen-Lung Wu, Deng-Fwu Hwang

**Affiliations:** a Department of Food Science, National Taiwan Ocean University, Keelung, Taiwan;; b Marine Fisheries Division, Fisheries Research Institute, Keelung, Taiwan;; c Planning and Information Division, Fisheries Research Institute, Keelung, Taiwan;; d Biodiversity Research Center, Academia Sinica, Taipei, Taiwan

**Keywords:** *Conus textile*, mitogenome, next-generation sequencing

## Abstract

The cone snail *Conus textile* belongs to the family Conidae. It is a kind of molluscivorous species. The complete mitochondrial DNA sequence was constructed by next-generation sequencing in this study. The mitogenome of *C. textile* is 15,765 bp in length, including 13 protein-coding genes, 22 tRNA genes, 2 ribosomal RNA genes and 1 control region. The base composition was 27.3% A, 37.9% T, 15.7% C and 19.1% G. The phylogenetic tree of *C. textile* with the other 6 *Conus* species and 15 Neogastropoda sea snails was built. It provides fundamental data for further research of phylogeny and biogeography with this genus.

The cone snails (*Conus*) are a species-rich genus of venomous marine gastropods. They inject their venom through a hypodermic needle-like radula harpoon that can penetrate deep into the dermis of their prey (Olivera, 1997). Their venoms consist of complex venoms composed mostly 100–250 disulfide-bridged peptides (Dobson et al. 2012). It’s a potent pharmacopoeia of individual bioactive peptide constituent, usually referred to as conotoxins or conopeptides (Bergeron et al. 2013). The cone snail *Conus textile* is a kind of molluscivorous sea snails (Röckel et al., 1995). We present the complete mitochondrial genome sequence of *C. textile* (Linnaeus 1758) in this study.

The specimens of *C. textile* (voucher no. 20141026-002; with Genbank accession no. KX155574) in this study were collected from north coast of Taiwan (25.203N, 121.695E). They are very common species in that area. The samples were deposited in Marine Toxins Lab., Department of Food Science, National Taiwan Ocean University, Taiwan. The total genomic DNA was extracted from muscle using magnetic bead technique with the KingFisher magnetic processors (ThermoFisher Scientific Inc., Worcester, MA). The raw next-generation sequencing reads generated from MiSeq sequencer (Illumina, San Diego, CA) were *de novo* assembled and reference mapping was conducted by commercial software (Geneious V9, Auckland, New Zealand) to produce a single circular form of complete mitogenome with about an average 24.3 coverage (1,340 out of 3,820,868 reads, 0.035%). The complete mitochondrial genome of *C. textile* is 15,765 bp in size, including 13 protein-coding genes, 22 tRNA genes, 2 ribosomal RNA genes (*12S* and *16S rRNA*) and 1 control region. The overall base composition of *C. textile* is 27.3% for A, 37.9% for T, 15.7% for C and 19.1% for G. The protein coding rRNA and tRNA genes of *C. textile* mitogenome were predicted by using MITOS (Bernt et al. 2013) and tRNAscan-SE (Schattner et al. 2005).

We used MEGA 6 (Tamura et al. 2013) to construct the phylogenetic relationships of the *C. textile* and related families by Neighbor-joining method with 1,000 bootstrap replicates based on the 13 protein-coding genes and 2 ribosomal RNA genes of the other 21 complete mitochondrial genomes of Neogastropoda sea snails, which are reported in Genbank of NCBI database. Bootstrap support values were relatively high, with 13 nodes having values >95% and 8 nodes demonstrating 100% bootstrap support (Figure 1). *C. textile* was grouped together with other six *Conus* species from the family Conidae. The lineages of Conidae strongly supported in this report and agreed with previous studies (Bouchet et al. 2011; Puillandre et al. 2014).

**Figure 1.**
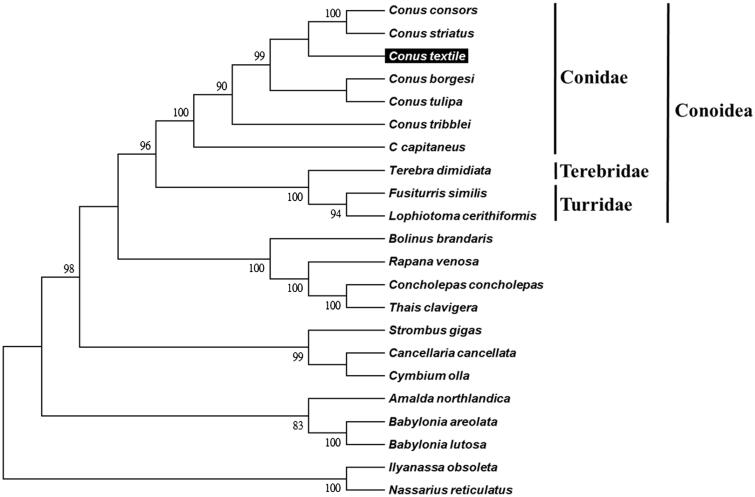
Phylogenetic tree generated using the neighbor-joining method based on complete mitochondrial genomes. *C. consors* (KF887950), *C. striatus* (KX156937), *C. textile* (KX155574), *C. borgesi* (EU827198), *C. tulipa* (KR006970), *C. tribblei* (KT199301), *C. capitaneus* (KX155573), *Terebra dimidiate* (EU827196), *Fusiturris similis* (EU827197), *Lophiotoma cerithiformis* (DQ284754), *Bolinus brandaris* (EU827194), *Rapana venosa* (KM213962), *Concholepas concholepas* (JQ446041), *Thais clavigera* (DQ159954), *Strombus gigas* (KM245630), *Cancellaria cancellata* (EU827195), *Cymbium olla* (EU827199), *Amalda northlandica* (GU196685), *Babylonia areolata* (HQ416443), *B. lutosa* (KF897830), *Ilyanassa obsoleta* (DQ238598) and *Nassarius reticulatus* (EU827201).
